# Perception of picky eating among children in Singapore and its impact on caregivers: a questionnaire survey

**DOI:** 10.1186/1447-056X-11-5

**Published:** 2012-07-20

**Authors:** Daniel YT Goh, Anna Jacob

**Affiliations:** 1 Department of Paediatrics, Head & Senior Consultant, Division of Paediatric Pulmonary & Sleep, University Children’s Medical Institute, National University Hospital, 5 Lower Kent Ridge Road, Singapore 119074, Singapore; 2 Department of of Paediatrics, Yong Loo Lin School of Medicine, National University of Singapore, Singapore, Singapore; 3 Nutrition Science and Communications, Abbott Nutrition International, Singapore, Singapore

**Keywords:** Child, Child preschool, Family health, Feeding behaviour, Singapore

## Abstract

**Background:**

Picky eating is relatively common among infants and children, often causing anxiety for parents and caregivers. The purpose of this study was to determine the key aspects of picky eating and feeding difficulties among children aged 1 to 10 years in Singapore and the impact on their parents or caregivers.

**Methods:**

In this survey, 407 parents or grandparents who are the primary caregivers of children aged 1 to 10 years in Singapore were interviewed via telephone using a structured questionnaire of 36 questions. Respondents were randomly selected from the Singapore Residential Telephone Directory to meet a pre-set interlocked quota of race, sex, and age to represent the population. Quantitative data collected included demographics, body weight and height, respondents’ perceptions of the duration of picky eating, the child’s eating habits and perceived health status, respondents’ attitudes towards picky eating, coping strategies and the impact on family relationships. Bonferroni z-test and *t*-test were used to indicate significance across groups or demographics, while Pearson correlation coefficient was used to measure the strength of association between variables.

**Results:**

One-half of the respondents reported that the child was ‘all the time’ (25.1%) or sometimes (24.1%) a picky eater. When aided with a list of typical behaviours, the respondent-reported prevalence of picky eating or feeding difficulties occurring ‘all the time’ increased to 49.6%. The highest number of respondents first noticed the child’s picky eating behaviours or feeding difficulties as early as 1 year (20.0%). Children 3 to 10 years [*p* = 0.022], children of professional respondents (*p* = 0.019), and children with a family history of picky eating (*p* = 0.03) were significantly more likely to be picky eaters. Overall, all ‘picky eating’ and all ‘feeding difficulty’ behaviours occurring ‘all the time’ were significantly associated with caregiver stress when feeding (*p* = 0.000026 and *p* = 0.000055, respectively) and with a negative impact on family relationships (*p* = 0.011 and *p* = 0.00000012, respectively).

**Conclusions:**

The perceived prevalence and duration of picky eating behaviours and feeding difficulties are high. The impact on the respondent and family relationships appears to be significant in Singapore. Parental concerns about picky eating should be adequately assessed and managed in routine clinic consultations.

## Background

Picky eating is relatively common among infants and children [1], often causing anxiety for parents and caregivers [2,3]. Picky eating is, however, not clearly defined; the term ‘picky eating’ has been described as consumption of an inadequate variety of foods [3]. The ICD-10 describes ‘feeding disorder of infancy and childhood’, encompassing ‘difficulty (in) feeding’, as generally involving food refusal and extreme faddiness in the presence of an adequate food supply, a reasonably competent caregiver, and the absence of organic disease (F98.2) [4].

Picky eating behaviours include rejection of certain types of food, acceptance of only certain foods, unwillingness to try new foods (food neophobia), limited intake of some food groups and strong food preferences [5]. Picky eating in early childhood can be related to eating disorders in adolescence and early adulthood [6], and can occur in normally developing children as well as in those with medical or developmental disorders [7]. Behavioural feeding disorders may be associated with suboptimal development [8], and some children with food refusal and picky eating have poor weight gain [3]. Importantly, parent’s perceptions of a child’s weight and eating habits may affect a child’s development and future lifestyle [9].

The prevalence of picky eating is difficult to ascertain, but has been reported to be as high as 50% in children aged 19 to 24 months in a study from the USA [10]. Between 20% and 60% of parents state that their young children are not eating optimally [8].

There is little information on picky eating and feeding difficulty in Asia. Therefore, this study was performed to determine the key aspects of picky eating and feeding difficulties among children aged 1 to 10 years in Singapore and the impact on caregivers.

## Methods

Specific objectives were to determine the local perceived prevalence of picky eating/feeding difficulties; parents’ and caregivers’ understanding of feeding difficulties; how parents and caregivers handle feeding difficulties; factors that influence feeding difficulties; and impact of feeding difficulties on the children and family relationships.

In this study, picky eating was characterised by fussiness about eating certain foods and fussy meal time behaviours, while feeding difficulty was characterised by sensory food aversion, fear of eating and insufficient intake.

### Participants

Inclusion criteria were a parent with a child aged 1 to 10 years or a grandparent who was the primary caregiver of a child aged 1 to 10 years. Exclusion criteria were chronic illnesses that impacted the child’s eating habits such as anorexia, gastro-oesophageal reflux disease, oesophagitis, food allergies and lactose intolerance.

### Design

In this questionnaire survey administered by telephone, parents or grandparents of children aged 1 to 10 years in Singapore were interviewed between 12 May and 16 June 2010 by trained interviewers. Respondents were randomly selected from the Singapore Residential Telephone Directory. The sample satisfied a pre-set interlocked quota of race, sex, and age to closely reflect the demographics of Singapore children aged 1 to 10 years.

407 respondents were interviewed by telephone and completed a structured questionnaire. Topics included demographic data, body weight and height, respondents’ perceptions of the duration of picky eating, the child’s eating habits and perceived health status, respondents’ attitudes towards picky eating, coping strategies and the impact on family relationships.

The interview took approximately 15 to 20 minutes to complete 36 questions. The questionnaire consisted of mostly closed ended categorical questions with listed options for the respondents to choose from (Additional file 1: Appendix). The main questions measured the behaviour, attitudes and perceptions of the respondents on picky eating and child feeding practices. A rating scale of 1 to 4 was used, where 1 = never, 2 = rarely, 3 = sometimes and 4 = all the time. Other categorical questions, for example “Do you think your child is a picky eater?”, were answered by ‘yes’, ‘no’, ‘sometimes’ or ‘in the past’. Only 6 questions were open-ended numeric questions covering the estimated height and weight of the child, the age of onset and duration of picky eating behavour and the number of meals and snacks taken per day. As the survey was quantitative, no detailed discussions were conducted.

A positive response of ‘all the time’ to any of the questions on picky eating behaviours qualified as picky eating. A positive response of ‘all the time’ to any of the questions on feeding difficulty behaviours qualified as feeding difficulty.

A series of statements relating to caregiver stress when feeding the child were asked and the respondents were requested to rate the degree of agreement based on a 5-point rating scale. Respondents rating the statement ‘agree’ or ‘strongly agree’ were considered to be experiencing stress.

### Statistical analysis

The margin of error calculated at the 95% confidence level for the sample size of 407 was 4.86% using the formula:

(1) $$\text{error}=1.96*\surd \left(\text{p}\left(1-\text{p}\right)/\text{n}\right)$$

where p is the observed percentage and n is the sample size.

For comparing proportions, Bonferroni z-test and *t*-test were used to indicate significance across groups or demographics, while Pearson correlation coefficient was used to measure the strength of association between variables.

## Results

### Respondents’ and Children’s characteristics

The respondents were most frequently the child’s mother (67.3%), followed by the father or grandmother, with 83.0% of the respondents being women (Table 1). The most common occupation was homemaker (37.6%), followed by professionals and other white-collar workers. Most respondents were educated to secondary (37.3%) or tertiary (47.5%) levels.

**Table 1:** Demographic characteristics of respondents and children

**Respondent profile (%)**		**Child profile (%)**	
Relationship to child		Sex	
Mother	67.3	Male	53.8
Father	15.7	Female	46.2
Grandmother	15.7	Age group (years)	
Grandfather	1.2	1-2	32.7
Occupation		3-5	32.9
PMEB	28.7	6-10	34.4
Other white collar	19.7	Race	
Blue collar	7.4	Chinese	64.4
Housewife	37.6	Malay	16.7
Unemployed	2.9	Indian/other	18.9
Other	3.7		
Education			
None	2.0		
Primary	13.3		
Secondary	37.3		
Tertiary (polytechnic)	19.7		
Tertiary (university)	27.8		

PMEB = professionals, managers, executives and businessmen.

Among the children, 53.8% were boys and 46.2% were girls. The age groups surveyed were 1 to 2 years, 3 to 5 years and 6 to 10 years, with a similar number of children in each age group (Table 1). The children were predominantly Chinese (64.4%). The child’s primary carer was usually a parent or grandparent (78.4%).

### Perceived prevalence of picky eating

Almost one-half of the respondents spontaneously reported that the child was always (25.1%) or sometimes (24.1%) a picky eater. Even though it was not statistically significant (*p* = 0.546), slightly more Chinese respondents (27.5%) felt that the child was a picky eater than did Malay (20.6%) or Indian respondents (20.8%).

Breakdown by age showed that the 3 to 5 years and 6 to 10 years age groups were significantly more commonly reported as picky eaters (29.9% and 25.0%, respectively) than were the 1 to 2 years age group (20.3%) [*p* = 0.022]. In addition, children of respondents who were professionals (34.2%) were significantly more likely to be perceived as picky eaters than respondents from other occupations (16.3% to 30.0%) [*p* = 0.019], as were children with a family history of picky eating (36.5%) when compared with children from families without a picky eating history (19.5%) [*p* = 0.030].

**Figure 1:**
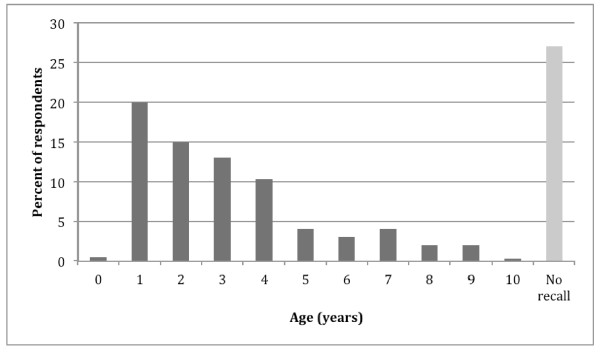
Age at first presentation of picky eating (n = 370).

**Figure 2:**
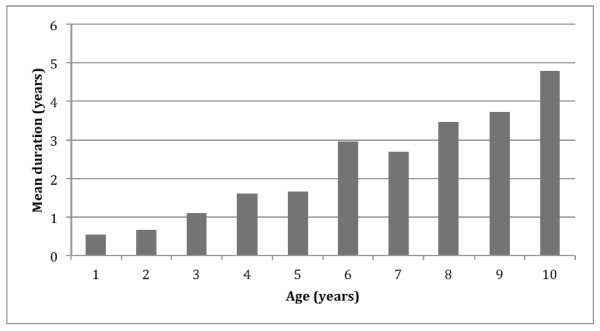
Mean duration of picky eating by age.

The highest number of respondents first noticed the child’s picky eating behaviours or feeding difficulties as early as 1 year (20.0%), with age having an almost linear correlation with first showing of these behaviours (Figure 1). Overall, the mean duration of picky eating was reported to be 0.6 years in a 1-year-old child, 1.1 years in a 3-year-old, 3.0 years in a 6-year-old and 4.8 years in a 10-year-old (Figure 2).

When the typical ‘picky eating’ and ‘feeding difficulty’ behaviours were explored by item, the prevalences of picky eating and feeding difficulties occurring ‘all the time’ were 47.4% and 15.2%, respectively, while the prevalence of any of either behaviour was 49.6%; 13.0% of children were reported to have both picky eating and feeding difficulties.

The most common typical behaviours of a picky eater among the group responding ‘always’ included eating slowly or holding food in the mouth (14.3%), refusing food, particularly fruit and vegetables (14.0%), eating sweets and fatty foods instead of healthy foods (13.3%), not liking to try new foods (12.0%), eating snacks instead of meals (11.1%) and accepting only a few types of food (12.0%). The prevalence of feeding difficulties occurring ‘always’ was reported as follows: not liking the texture of certain foods (6.6%), eating very little food (9.6%) and fear of certain foods due to a previous bad experience (4.4%).

### Perceived health status

The respondents generally perceived the child’s height and weight to be normal (63.6% and 75.2%, respectively). Only 7.8% and 13.3% of respondents felt that the child was very short or short, and underweight or of low weight, respectively. Only 2.5% of the respondents reported that the child was overweight. There was a significant correlation for respondents’ perceptions of the child’s weight and body mass index (BMI) when the BMI was within the normal range (p = 0.031; Table 2), but there was no correlation between respondents’ perceptions of the child’s weight and BMI when the BMI was either low or high.

**Table 2:** Correlation between respondents’ perception of their child’s weight and body mass index

**Body mass index**	**Correlation coefficient**	** *p* Value**
<14	0.076	0.576
14 to <19	0.180	0.031*
≥19	−0.177	0.114

*Significant correlation.

Overall, spontaneous report of picky eating was significantly associated with the respondents’ perception that the child was not energetic and healthy (*p* = 0.048). When explored, only ‘not liking the texture of certain foods’ occurring ‘all the time’ when compared with ‘never’, ‘rarely’ and ‘sometimes’ was significantly associated with the respondents’ perception of a decrease in the child’s health and activity levels (*p* = 0.0001). Similarly, ‘fear of certain foods due to a bad experience’ (*p* = 0.025) and ‘throwing tantrums at mealtimes’ (*p* = 0.019) were significantly associated with the caregivers’ perception of an increase in the child’s level of sickness and tiredness.

### Attitudes and coping mechanisms

**Figure 3:**
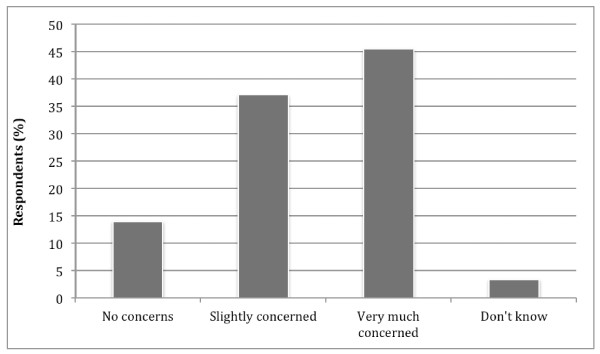
Respondents’ concerns about their child’s picky eating behaviour.

Nearly half of the respondents (45.5%) reported that they were ‘very much concerned’ that the child was a picky eater and a further one-third (37.1%) were ‘slightly concerned’ (Figure 3). Respondents who cared for the child themselves tended to be more concerned about the child’s eating habits than a relative or ‘other’ caregiver (54.9% versus 30.1–50.0%). The most common concerns about the consequences of picky eating behaviours were the child’s physical and mental development (83.3% and 54.5%, respectively).

Children tended to eat a mean of 2.9 meals per day (range, 1–6) and had a mean of 1.9 snacks per day (range, 0–5). A significant negative correlation was observed between the number of meals and the number of snacks eaten per day for all respondents (R^2^ = 0.01) and this was greater for those who spontaneously reported that the child is a picky eater all or some of the time (R^2^ = 0.06).

Most respondents felt responsible for ensuring that the child was eating the right types of food (89.2%), ensuring that the child did not have too many fatty foods, sweets and junk foods (88.7%) and encouraging the child to eat with the family (89.9%). Most respondents personally made sure that the child had enough food at every meal (94.6%) and pressured them to always eat all the food on the plate (86.2%). To ensure that the child received sufficient nutrition, respondents tended to ensure the meals included vegetables, fruits and meats (62.7%), watch what the child eats (46.4%), plan or supervise the child’s meal (41.8%) and give the child milk from a bottle (40.0%).

The respondents used a variety of coping strategies (‘all the time’ and ‘sometimes’), including modifying the texture to make food easy to eat (65.6%), allowing television viewing at mealtimes (62.4%) and presenting food in an age-appropriate manner (use of coloured cups or bottles; 52.8%).

Certain attitudes and perceptions of parents or grandparents of children with feeding difficulties were significantly associated with the reported persistent prevalence of feeding difficulties (Table 3). Of these, pressure to eat such as ‘raising the voice and threatening the child until the food is finished’ (*p* = 0.000003) and ‘making the child eat when not hungry’ (*p* = 0.001) were most positively associated with persistent behaviour, while ‘deciding the type and quantity of food’ for the child was negatively associated (*p* = 0.005). Of the coping strategies, persistent prevalence was significantly associated with ‘consulting a doctor about the child’s eating habits’ (*p* = 0.001), ‘allowing a maid/caregiver to feed the child’ (*p* = 0.015) and ‘giving the child milk from a bottle’ (*p* = 0.002).

**Table 3:** Association of respondents’ attitudes and perceptions of child feeding with reported prevalence of feeding difficulties

**Respondents’ attitudes and perceptions of child feeding (occurring ‘all the time’ compared with ‘never’, ‘rarely’ and ‘sometimes’)**	**Feeding difficulties occurring ‘all the time’**		
	** *p* Value (95% confidence interval)**	**Percent decrease**	**Percent increase**
Pressure to eat			
I have to raise my voice and threaten my child until he/she finishes the food	0.000003 (0.12–0.42)		27.2
If my child says “I’m not hungry”, I try to get him/her to eat anyway	0.001 (0.04–0.27)		15.7
Responsibility			
I decide if my child is eating the right types and quantity of food	0.005 (−0.18– -0.03)	10.4	
Coping strategy			
I consult a doctor about my child’s eating habits.	0.001 (0.06–0.44)		24.8
I let my maid or other caregiver feed my child.	0.015 (0.01–0.24)		12.4
My child drinks from a milk bottle.	0.002 (0.04–0.20)		11.6

### Family history and relationships

Approximately one-third of respondents (31.0%) had other family members that they considered to be picky eaters. Respondents who perceived the child to be picky eaters tended to have a family history of picky eating (45.1%) [*p* = 0.030].

Most respondents enjoyed feeding the child (54.5%) and felt relaxed at mealtimes (70.8%). Ten of the 12 picky eating behaviours occurring ‘all the time’ were significantly associated with respondents’ reports of stress during feeding; 9 of the 12 picky eating behaviours occurring ‘all the time’ were significantly associated with a negative impact on family relationships. All 3 ‘feeding difficulty’ behaviours occurring ‘all the time’ were significantly associated both with reports of stress in the caregivers when feeding and with family relationships being affected (Table 4).

**Table 4:** Association of picky eating and feeding difficulty behaviours with feelings of stress and with a negative impact on family relationships

	**I feel stressed and frustrated when feeding my child (selected ‘strongly agree’)**		**My family relationships are affected because of the stress of feeding my child (selected ‘strongly agree’)**	
**Behaviour (occurring ‘all the time’ compared with ‘never’, ‘rarely’ and ‘sometimes’))**	***p*Value (95% confidence interval)**	**Percent increase**	***p*Value (95% confidence interval)**	**Percent increase**
Picky eating behaviours				
Complain about what is served	0.001	24.2	0.000003	20.2
	(0.0664–0.4176)		(0.0491–0.3539)		
Refuse food especially vegetables/fruits	0.001	18.1	0.0001	12.9	
	(0.0576–0.3046)		(0.0331–0.2256)		
Refuse food like meats	0.0004	18.3	0.003	11.9	
	(0.0339–0.3314)		(0.0037–0.2333)		
Push, hide or throw food during mealtime	0.021	28.2	0.051	3.8	
	(0.0015–0.5635)		(−0.1200–0.1955)		
Eats the same food for all the meals	0. 052	12.0	0.225	4.8	
	(0. 0029–0.0237)		(−0.0285–0.1240)		
Accept only a few types of food	0.009	16.3	0.0332021	8.6	
	(0.0318–0.2938)		(−0.0077–0.1799)		
Not like to try new food	0.059	11.6	0.021	8.6	
	(−0.0089–0.2417)		(−0.0077–0.1799)		
Eat slowly or hold food in the mouth	0.0003	19.6	0.071	6.6	
	(0.0721– -0.3194)		(−0.0147–0.1471)		
Eat sweets and fatty foods instead of healthy foods	0.009	15.6	0.00002	13.8	
	(0. 0317–0.2802)		(0.0374–0.2392)		
Eat snacks instead of meals	0.0004	21.5	0.00003	14.7	
	(0.0728–0.3563)		(0.0343–0.2605)		
Throw tantrums at mealtimes	0.000000001	46.8	0.0000001	17.2	
	(0.2686–0.6683)		(0.0082–0.3353)		
Prefer drinks to food	0.005	15.6	0.0001	11.7	
	(0.0317–0.2802)		(0.0205–0.2135)		
Feeding difficulty behaviours					
Not like the texture of certain foods	0.00002	32.3	0.0003	14.8	
	(0.1331–0.5137)		(0.0006–0.2961)		
Fear certain foods due to a bad experience previously	0.001	31.6	0.00008	18.4	
	(0.0840–0.5479)		(−0.0094– -0.3767)		
Eat very little	0.038	12.6	0.006	9.0	
	(−0.0153–0.2673)		(−0.0166–0.1969)		

## Discussion

This study attempts to provide an overview of picky eating and feeding difficulties among children aged 1 to 10 years in Singapore and the impact on the children’s parents or caregivers. The study was conducted as a questionnaire survey among a representative sample of the Singapore population.

The spontaneous caregiver-reported prevalence of picky eating in this study was 49.2%, which is similar to that of 50% reported by Carruth et al in their cross-sectional survey [10]. Other studies have found rates ranging from 17% in China to 29% in Canada [11-14]. When exploring typical behaviours, 49.6% of respondents’ children exhibited at least one ‘picky eating’ or ‘feeding difficulty’ behaviour ‘all the time’.

The most common behaviours occurring ‘all the time’ were eating slowly or holding food in the mouth; refusing food, particularly fruit and vegetables; eating sweets and fatty foods instead of healthy foods; food neophobia; eating snacks instead of meals and accepting only a few types of food. Wright et al found that eating a limited variety and preferring drinks to food were the most prevalent problem behaviours [3], while Jacobi et al. reported that picky eaters ate fewer foods and were more likely to avoid vegetables [5]. Mascola et al found that picky eaters were more likely to consume a limited variety of foods, required food prepared in specific ways, expressed stronger likes and dislikes for food, and had tantrums when denied foods [2]. Interestingly, in this study, respondents who reported that the child was not a picky eater were more likely to report picky eating behaviours of 'eating slowly' or 'eating sweets instead of healthy foods' (occurring 'all the time' and 'sometimes'). This possibly reflects greater cultural acceptance of these picky eating behaviours as normal in Singapore, thus overlooking potential consequences.

Although some studies have shown that picky eating does not affect health or weight gain [11,14], two studies found that children with eating problems gained less weight than children without eating problems [3,15]. Reduced intakes of energy, carbohydrate, fat and protein have been found to be evident among children with picky eating and feeding difficulties [15,16], although most children with problem eating achieve normal growth [3]. There was no evidence that the children in this study were smaller than expected for their age, but further study is needed to ascertain the impact of eating problems on childhood development.

In this study, the older age groups were more likely to be picky eaters. The mean duration of picky eating by age suggests that picky eating may be a persistent and chronic problem in childhood, as reported in the study by Mascola et al. [2].

Picky eating caused the respondents considerable concern with nearly half being ‘very much concerned’; the concerns were predominantly about the child’s physical and mental health. This is in agreement with the study by Mascola et al that found that picky eating is of considerable parental concern [2]. It is interesting to note that some of the respondents’ attitudes and perceptions towards child feeding (deciding what the child will eat, raising the voice, threatening the child and making the child eat even when not hungry) were significantly associated with the reported prevalence of ‘feeding difficulty’ behaviours. However, it is not clear whether the associations noted are the cause or the result of the mealtime behaviours.

One-third of respondents had other family members who were picky eaters, and those who perceived the child to be picky eaters tended to have a family history of picky eating. Most ‘picky eating’ and all ‘feeding difficulty’ behaviours appear to be significantly associated with respondents’ stress when feeding the child and with a negative impact on family relationships.

The respondents used a variety of coping strategies, including modifying the texture to make food easy to eat, allowing television viewing at mealtimes and presenting food in an age-appropriate manner (use of coloured cups or bottles). Other strategies cited involved consulting a doctor about the child’s eating habits, allowing a maid/caregiver to feed the child and giving the child milk in a bottle. Nearly one-third of respondents (29.2%) consulted a doctor about the problem of picky eating/feeding difficulty. Thus, clinic visits provide an opportunity for clinicians to assess the problem, provide support and guidance to parents, exclude any underlying pathology, and initiate appropriate management.

### Study limitations and future research recommendations

While this study provides insight into the attitudes of parents with children who are picky eaters in Singapore, the study investigated their perceptions of picky eating and it’s impact on the child’s health and family relationships. All data were caregiver-reported, with no independent measurement of the children’s mealtime behaviours or caregivers’ stress. Further studies are needed to fully understand the regional and ethnic variations in attitudes and coping strategies, as well as the impact on the child, caregiver and other family members.

## Conclusion

Caregivers of children who are picky eaters or have feeding difficulty behaviours were concerned about the consequences of picky eating behaviours on the children’s physical and mental development. Picky eating behaviours caused the respondents much stress when feeding their children. One-third of parents consult their doctor about their child’s eating behaviours; during clinical consultations, parental concerns about picky eating should be adequately assessed and managed. Clinicians can help to guide parents on the best approaches to achieving good nutrition for children who are picky eaters.

## Competing interests

Daniel YT Goh has no competing interests. Anna Jacob is an employee of Abbott Laboratories (Singapore) Pte Ltd.

This study was supported by an education grant from Abbott Nutrition, a division of Abbott Laboratories (Singapore) Pte Ltd.

## Authors’ contributions

DG contributed the concept and design of study, analysis and data interpretation, revisions, review and approval of the manuscript. AJ contributed the concept and design of study, data collection, compilation, analysis and interpretation of data and preparation of the manuscript. Both authors read and approved the final manuscript.

## Authors’ information

DG, MBBS(S'pore), MMed(Paeds), FRCPCH(UK), FCCP(USA), FAMS, is an Associate Professor and Head of the Department of Paediatrics, Head & Senior Consultant, Division of Paediatric Pulmonary & Sleep, University Children’s Medical Institute, National University Hospital and Yong Loo Lin School of Medicine, National University of Singapore, Singapore.

AJ, MSc Nutrition and Dietetics, BSc Food Service Management and Dietetics, is Senior Manager of Nutrition Science and Communications, Abbott Nutrition International, Singapore.

## Supplementary Material

Additional file 1 **Appendix.** Child Eating Habits Survey 2010. Click here for file
