# Supplementary material for: Perception of picky eating among children in Singapore and its impact on caregivers: a questionnaire survey
Source: Asia Pac Fam Med. 2012 Jul 20;11(1):5. doi: 10.1186/1447-056X-11-5 (PMC3477031; doi:10.1186/1447-056X-11-5)
Supplement: Additional file 1 — Appendix. Child Eating Habits Survey 2010. [file 1447-056X-11-5-S1.docx]

**Appendix. Child Eating Habits Survey 2010**

***Screening/Background***

*Section A: Child’s Eating Habits*

A1. In your opinion, what is picky eating? [Unprompted; multiple answer]

1 Refuses many foods

2 Refuses fruit

3 Refuses vegetables

4 Refuses meat/fish/poultry and other protein-rich foods

5 Refuses food of particular texture

6 Refused new foods

7 Eats the same few foods only

8 Eats limited amount (quantity) of food

9 Others, specify: ____________________________________

A2. Do you think your child is a picky eater?

1 Yes

2 No

3 Sometimes

4 In the past

A3. I will be reading out typical behavior of a picky eater. Please let me know if each of them applies to your child and how frequently it does.

On a scale of 1-4 does your child…

*Picky eating behaviours*

a. Complain about what is served

b. Refuse food especially vegetables/fruits

c. Refuse food like meats

d. Push, hide or throw food during mealtime

e. Eats the same food for all the meals

f. Accept only a few types of food

g. Not like to try new food

h. Eat slowly or hold food in the mouth

i. Eat sweets and fatty foods instead of healthy foods

j. Eat snacks instead of meals

k. Throw tantrums at mealtimes

l. Prefer drinks to food

*Feeding difficulties*

m. Not like the texture of certain foods

n. Fear certain foods due to a bad experience previously

(Only read if necessary: eg. Fish with bones, fish balls or nuts due to choking)

o. Eat very little

On a scale of 1-2…

p. Is your child energetic and healthy?

q. Or is your child tired often and/or falls sick often?

*Ask, if child is a picky eater*

A4. At what age did you first notice your child’s picky eating behaviour (mentioned above)?

A5. How long have you observed your child’s picky eating behaviour (mentioned above)?

*Ask all*

A6. How concerned are you/would you be if your child is a picky eater?

1 No concerns

2 Slightly concerned

3 Very much concerned

4 Don’t know

A7. What are/would be your concerns for your child if he/she is a picky eater? [multiple answer]

1 Physical development of child

2 Mental development of child

3 Others, please specify: ______________________________

A8. Were you or any other family members a picky eater?

1 Yes

2 No

3 Don’t know

A9. On a scale of 1-5 are you confident that your child is getting enough nutrients from his/her food and drinks?

A10. How often does your child eat per day? [A main meal is defined as breakfast, lunch or dinner and snacks* are defined as light food provided in between main meals.]

* May include food or beverages only.

***Section B: Parent’s/Grandparent’s Attitude To & Perception of Child’s Feeding***

As I read out a few statements, please rate each one on a scale of 1 to 4, where 1 means never, 2 rarely, 3 sometimes and 4 all the time.

*B1. Responsibility*

a. I decide if my child is eating the right types and quantity of food.

b. I am responsible for feeding my child at home.

*B2. Food and Eating Rules*

a. I have to be sure that my child does not eat too many high fat foods, sweets (candy, ice cream, cake) and junk foods.

b. I encourage my child to eat with the family.

c. I offer my child his/her favourite foods in exchange for good behavior.

*B3. Pressure to eat*

a. My child should always eat all of the food on his/her plate.

b. I have to make sure that my child eats enough at every meal and snack.

c. I have to raise my voice and threaten my child till he/she finishes the food.

d. My child has to be fed during every meal.

e. If my child says “I’m not hungry”, I try to get him/her to eat anyway.

*B4. Coping strategies*

a. I turn on the TV during meal times to make him/her eat.

b. I modify the texture of food for my child – cut the food up into small portions, mince, blend or cook until very soft, etc.

c. I serve food or drinks on cups/ colored cups or bottles.

d. My child drinks from a milk bottle.

e. My child’s food is prepared differently from the rest of the family.

f.

i) I use a high chair to restrict his/her movement in order to finish the food served (for children < 3 yrs old)

OR

ii) I insist that my child sits at the dining table for meals (for children > 3 yrs old)

g. I feed my child the same way my parents did when I was young.

h. I let my maid / other caregiver feed my child

i. I consult a doctor about my child’s eating habits.

B5. What do you do to ensure that the child gets the nutrition he/she needs? [Unprompted; multiple answer]

1 Watch what he/she eats

2 Plan/supervise his/her meal

3 Ensure his/her meals include vegetables, fruits & meats

4 Give him/her vitamin and mineral supplements

5 Ensure that he/she has her milk feed

6 Avoid snacks

7 Avoid fast food

8 Avoid oily food

9 Feed him / her

10 Force or coerce him / her

11 Nothing

12 Others, specify: ______________________________

B6. Where do you get the information on child nutrition and amount of servings should a child eat? [Unprompted; multiple answer]

1 TV (ads/programmes)

2 Radio (ads/programmes)

3 Newspapers (ads/articles)

4 Magazines (ads/articles)

5 Health Journals/Books

6 Posters/ pamphlets at health clinics

7 Internet

8 Friends/Relatives/Colleagues

9 Doctors/Healthcare Professionals

10 Dietitian/Nutritionist

11 Seminars/Talks/Exhibitions

12 Others, specify: _______________________

B7. Please rate from 1-5 the following statements when you feed your child?

a. I like feeding my child

b. My child and I enjoy/feel relaxed during mealtimes

c. I feel stressed and frustrated when feeding my child

c. I usually pass the feeding job to another caregiver (e.g. maid)

d. My family relationships are affected because of the stress of feeding my child

***Section C: Demographics***
